# Does Loop Electrosurgical Excision Procedure of the Uterine Cervix Affect Anti-Müllerian Hormone Levels?

**DOI:** 10.1155/2014/875438

**Published:** 2014-02-23

**Authors:** Martha M. Sklavos, Cassandra N. Spracklen, Audrey F. Saftlas, Ligia A. Pinto

**Affiliations:** ^1^Human Papillomavirus Immunology Laboratory, Leidos Biomedical Research, Incorporated, Frederick National Laboratory for Cancer Research, Building 469, Room 111, 1050 Boyles Street, Frederick, MD 21702, USA; ^2^Department of Epidemiology, University of Iowa College of Public Health, 145 Riverside Drive, S471 CPHB, Iowa City, IA 52242, USA; ^3^Department of Epidemiology, University of Iowa College of Public Health, 145 Riverside Drive, S427 CPHB, Iowa City, IA 52242, USA

## Abstract

*Background*. A delayed time to pregnancy was recently reported for women who had a loop electrosurgical excision procedure (LEEP) to remove cervical intraepithelial neoplasia (CIN) grade 2 or 3. The objective of the current study was to determine if treatment of CIN with LEEP is associated with decreased levels of anti-Müllerian hormone (AMH), a marker of ovarian reserve. *Methods*. AMH levels were measured in 18 women treated with LEEP and 18 age-matched controls, who had colposcopy only and did not require LEEP. Cases and controls had their blood drawn at study entry time zero and again 6 months later. *Results*. The mean AMH level decreased significantly from baseline to follow-up; however, no significant differences were observed when stratifying by LEEP status, suggesting that both groups experienced a similar decrease in AMH levels during the follow-up period. Although women treated with LEEP had lower overall AMH levels than controls at both baseline and follow-up, these differences were not statistically significant. *Conclusion*. Overall, the delayed time to pregnancy observed in women treated with LEEP is likely not due to a LEEP-associated decrease in ovarian reserve as measured by AMH; thus, other mechanism are responsible for the delayed time to pregnancy associated with LEEP.

## 1. Introduction

In the United States (US), pap smears to detect precancerous cervical intraepithelial neoplasia (CIN) are an integral part of a woman's health care regimen. As a result of screening, women with biopsy confirmed CIN2 or CIN3 routinely undergo loop electrosurgical excision procedure (LEEP) to remove cervical dysplasia, equating to approximately half a million LEEP procedures in the United States each year (American Cancer Society/[1]). Because most women requiring LEEP are of child bearing age, preserving fertility is paramount. However, the impact of LEEP on fertility is not established. Although a few small studies from the early 1990s concluded that LEEP had no effect on future fertility, these studies were not designed to directly address this question and lacked information on potentially confounding factors such as a history of infertility [2–4].

In a recent study of time to pregnancy following LEEP and other cervical surgical procedures, Spracklen et al. reported that women with a history of LEEP were significantly more likely to require more than 12 months to conceive a pregnancy resulting in a live birth (odds ratio 2.47, 95% Confidence Interval 1.10–5.55) when compared to similar women with no history of cervical surgery [5]. While this finding suggests that LEEP is associated with reduced fertility, the underlying mechanisms responsible for this link have not yet been identified. Because LEEP is a common procedure, investigating underlying biological causes of a delayed time to conception of a viable pregnancy for women with a history of LEEP is of interest.

Anti-Müllerian hormone (AMH) is an established marker of ovarian reserve, which is tightly linked to female fertility. Therefore, measuring AMH levels before and after LEEP could determine if the procedure affects this vital component of female fertility. AMH is a small peptide hormone within the TGF-beta family that is currently used to diagnose subfertility/infertility, primary ovarian insufficiency (POI), and polycystic ovarian syndrome (PCOS), among other disorders. In addition, AMH is now the leading predictor of in vitro fertilization (IVF) success [3, 4, 6–8]. Recent literature has demonstrated that AMH is a more reliable measure of fertility than follicle-stimulating hormone (FSH) [4, 7–11]. Normal ranges of AMH are broad and the normal range defined specifically for the AMH assay used in this study is 1–8 ng/mL [12]. For healthy women of reproductive age, an AMH level less than 1 ng/mL signifies impaired fertility due to an inadequate ovarian reserve [12]. AMH can be measured at any point in the menstrual cycle as it does not appear to fluctuate significantly throughout the cycle, unlike FSH, which must be measured on day 3 of the menstrual cycle [6–8]. Because the only cells that produce AMH in women are the granulosa cells in the ovaries, AMH is not subject to the feedback mechanisms of the hypothalamic-pituitary-gonadal axis [6, 8]. In women, AMH levels rise just prior to puberty, remain elevated throughout a woman's peak reproductive years, then significantly decline to undetectable levels in the years prior to menopause, indicative of a loss of fertility [13, 14].

Elevated levels of cytokines resulting from endometriosis, sexually transmitted infections, pelvic inflammatory disease, and other gynecologic disorders have been reported to negatively influence fertility. We hypothesize that the nonspecific inflammation and associated cytokine production resulting from LEEP may cause indirect damage to the urogenital microenvironment, thereby impairing ovarian reserve [12, 15]. Inflammatory cytokines are capable of negatively affecting ovulation, hormones required for reproduction, sperm and egg quality, and implantation [12, 15, 16]. To investigate the potential causes for the described decrease in fertility after LEEP [5], we designed a study to compare levels of the fertility marker, AMH, in women of reproductive age who had LEEP (CIN2/3) and a similar group of women who did not require LEEP (<CIN2) following colposcopy.

## 2. Materials and Methods

### 2.1. Subjects

The subjects for this analysis participated in a longitudinal study to assess the impact of LEEP on the immunologic properties of cervical mucus. Of these subjects, only a subset was used for our current study assessing the effect of LEEP on AMH levels (Supplemental Figure 1 in Supplementary materials available online at http://dx.doi.org/10.1155/2014/875438). This study recruited women of reproductive age who had underwent colposcopy at the University of Iowa from 2009-2010. Written informed consent was obtained to permit collection and analysis of demographic data, clinical data, cervical secretions and blood samples. All protocols and informed consent procedures were approved by the University of Iowa Institutional Review Board. Women eligible to participate were 18–38 years old, were not pregnant, had no prior history of cervical surgery, had regular menstrual cycles of 21–35 days, and had no history of D&C, induced abortion, cervical dysplasia, cancer, HIV/AIDS, or autoimmune disease (e.g., rheumatoid arthritis, lupus, and multiple sclerosis). Samples were not collected from women who used oral steroids within the past 2 weeks or inhaled steroids within the past 24 hours; used emergency contraception in the past 30 days; douched within the past 48 hours; engaged in vaginal intercourse within the past 48 hours; currently had a vaginal or sexually transmitted infection other than HPV; became pregnant during the study; or were currently menstruating.

A total of 63 qualifying subjects who required a LEEP procedure (cases: CIN2/3) and 49 subjects who did not require cervical surgery (controls: <CIN2) enrolled in the study. A subset of the enrolled women (19 LEEP and 28 No LEEP controls) also consented to provide blood samples at baseline and at follow-up for future analysis. Cases and controls had their blood drawn at study entry time zero (baseline), which was just prior to the L EEP procedure for cases, and again 6 months later (follow-up). Blood was collected during the same phase of the menstrual cycle for each individual woman at both time points.

For this study, we selected all 19 available cases, defined as women who were treated with LEEP after colposcopy and 19 age-matched controls, who did not receive LEEP after colposcopy. Cases and controls were age-matched within 2 years because AMH levels are known to decrease with age following peak fertility [13, 17–19]. Five of 19 cases and 2 of 19 controls were smokers at study entry and through the follow-up period. Additionally, 18/19 cases and 15/19 controls were using hormonal contraception at study entry and continued to do so throughout the duration of the study. One case-control pair was eliminated because of an approximately 300% increase in AMH levels from baseline to follow-up measurements. All other subjects had less than a 60% change in AMH levels from baseline to follow-up. Reported analyses were performed on the remaining 18 cases and 18 age-matched controls.

### 2.2. AMH ELISA Assay

AMH was measured in the serum of all subjects using the sensitive (LOD = 0.09 ng/mL) Gen II AMH ELISA from Beckman Coulter, Inc. (United States) at Frederick National Laboratory for Cancer Research (FNLCR) (Frederick, MD) according to the manufacture's protocol. This assay is used clinically in Europe and in the US. Excellent intra- and interplate reproducibility has been reported in the literature for this assay [20]. The assay reproducibility was confirmed in the FNLCR lab where the assay consistently performed well with intra- and interplate variability of <10%. For this study, case and control samples were randomized and run in duplicate in three separate AMH assays.

### 2.3. Statistical Analysis

Univariate, bivariate, and stratified analyses were conducted to assess the change in AMH levels within the LEEP and No LEEP groups over the 6-month follow-up period. Paired *t*-tests were used to compare mean AMH levels from baseline to follow-up. Analysis of variance was performed to assess for differences in the mean change in AMH levels between case and control groups. Percent change in AMH levels between baseline and follow-up was calculated for all subjects. Although the estimated percent change in AMH in a healthy population of reproductive age over a 6-month period is 7.5% [21], the variability of the assay can range to almost 10%. For this reason, we defined ±9.9% change in AMH levels over the follow-up period as being equivalent to no significant percent change in AMH. All data analyses were conducted using SAS software, version 9.3 for Microsoft, SAS Institute Inc., Cary, NC, USA. Graphs were generated using GraphPad Prism 4.

## 3. Results and Discussion

### 3.1. Results

**Figure 1:**
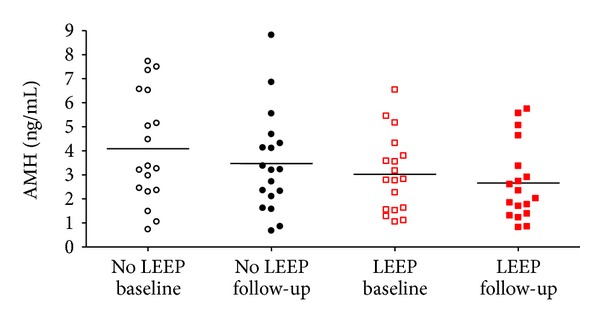
AMH levels at baseline and follow-up grouped by treatment status. Each data point on the graph represents a single AMH measurement. Each individual (No LEEP: *n* = 18; LEEP: *n* = 18) is represented by two data points, one at baseline and one 6 months later at follow-up. The black horizontal lines represent the mean value for each group. ○ No LEEP baseline, ● No LEEP follow-up, □ LEEP baseline, and ■ LEEP follow-up.

The mean AMH levels for all subjects by LEEP status are shown in Table 1. Mean levels of AMH declined significantly from baseline to the 6-month follow-up among all 36 subjects (3.56 versus 3.07 ng/mL, *P* = 0.009). When women were stratified based on LEEP status, both groups had a similar decrease in AMH, although the decrease was not statistically significant at the *P* < 0.05 level (LEEP, *P* = 0.07; No LEEP, *P* = 0.06). Women from the LEEP group had lower levels of AMH compared to the No LEEP group at both baseline (LEEP: 3.02 ng/mL versus No LEEP: 4.09 ng/mL) and follow-up (LEEP: 2.66 ng/mL versus No LEEP: 3.48 ng/mL), though these differences were not statistically significant. At the 6-month follow-up visit, two women in each group had AMH levels indicative of an impaired ovarian reserve (<1 ng/mL) (data not shown).  Figure 1 shows the AMH measurements for each subject at baseline and follow-up stratified by LEEP status.

**Figure 2:**
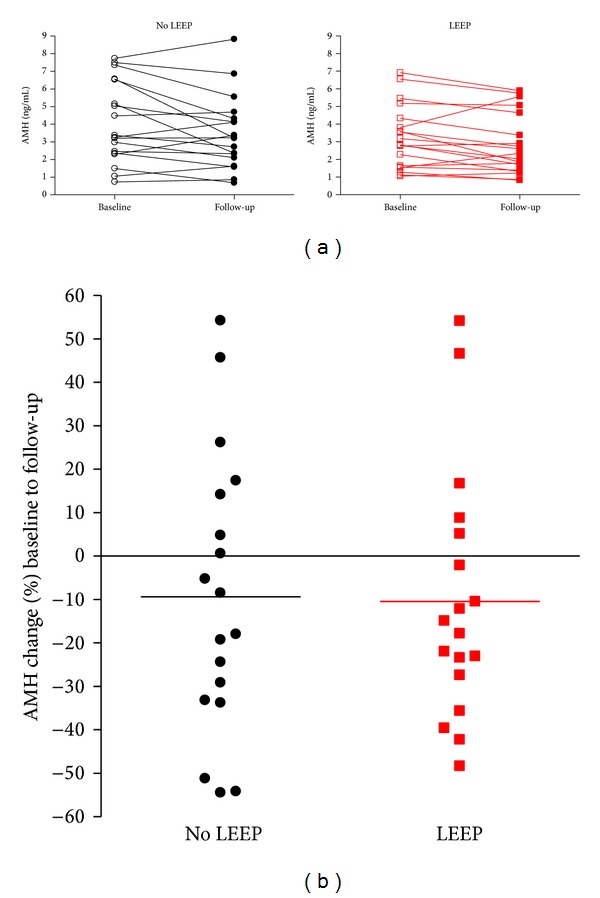
Change in AMH levels from baseline to follow-up. In (a), the two graphs, one graph for No LEEP subjects (*n* = 18) and another for LEEP subjects (*n* = 18), represent the two AMH measurements (baseline and follow-up) from each individual. Baseline and follow-up measurements are connected linearly to show the increase or decrease in AMH over the 6-month follow-up period. ○ No LEEP baseline, ● No LEEP follow-up, □ LEEP baseline, and ■ LEEP follow-up. In (b), the percent change in AMH levels between baseline and follow-up AMH measurements is plotted by treatment status for each subject. Each data point represents the percent change in AMH from baseline to follow-up for each subject, and the horizontal line represents the mean percent change for each treatment group. ● No LEEP and ■ LEEP.

Figure 2(a) displays the baseline and follow-up AMH levels for each subject. Overall, AMH levels decreased over the 6-month follow-up period for most women. Although a reduction in AMH over time is expected, several subjects' levels decreased in excess of the 9.9% expected based on age and assay variability [21]. On average, AMH levels in LEEP and No LEEP subjects decreased similarly (−10.47% versus −9.38%, resp.) (Figure 2(b)). There was not a significant difference in the overall decrease in AMH levels in women who had tissue removed by LEEP versus their age-matched controls who had no tissue removed; however, more subjects from the LEEP group had a notable decrease in AMH levels (>9.9%) compared to No LEEP subjects (12 versus 9) (Table 2). Similar numbers of LEEP and No LEEP subjects had no significant change in AMH levels (<±9.9%) (3 versus 4), though almost twice as many control subjects had an increase in AMH levels (>9.9%) when compared to case subjects (5 versus 3).

### 3.2. Discussion

In the first study to investigate underlying mechanisms of reduced fertility after LEEP, our findings suggest that AMH is not affected by LEEP and, therefore, another mechanism is responsible for the delayed time to pregnancy observed in women treated with LEEP. Furthermore, innate and environmental factors can cause the levels of AMH to vary broadly from woman to woman, even of the same age, so the fairly large range of AMH levels observed in our study is not surprising [17]. Over the 6-month follow-up period, the percent decrease in AMH levels in women who had LEEP versus those observed in age-matched controls was quite comparable and not statistically significant. These data suggest that LEEP does not significantly affect ovarian reserve.

An interesting observation from this small study is that the mean and median AMH levels for LEEP subjects, all of whom had been diagnosed with CIN2/3 cervical lesions, tended to be lower than those among control women who had less than CIN2 pathology and did not require LEEP. This is in agreement with our finding that women whose AMH levels increased over the 6-month follow-up period were less likely to be in the LEEP group (CIN2/3). There may be an increased risk of cervical disease for women with lower AMH levels since mounting evidence demonstrates the anticancer effects of AMH in vitro and in vivo [13]. It is possible that AMH may play a role in cancer control in humans [13]. In recent years, AMH has been investigated as an anti-cancer agent, in addition to its better known role as a predictor of female fertility.

Because pathology reports and HPV DNA information were unavailable, further studies are needed to investigate the possibility of a direct association between HPV-associated cervical disease and AMH. Small sample size is another limitation of the current study. To address these limitations we are conducting a larger study to more directly investigate if lower AMH levels can serve as a risk factor for cervical disease.

Factors known to negatively affect AMH levels include smoking, chemotherapy, radiation, and any surgery removing or disturbing the ovaries [22–25]. It is largely reported that hormonal contraceptives do not have a significant effect on AMH levels and the levels can be measured at any time during the menstrual cycle; however, these issues are still debated in the literature.

Our study was controlled for any potential AMH flux as a result of cycling as AMH was measured during the same phase of the menstrual cycle for each subject's baseline and follow-up visits. However, AMH was not measured during the same phase of the menstrual cycle for all case and control subjects on the whole. Factors that may lead to an increase in AMH production include PCOS, granulosa cell ovarian tumors, smoking cessation, and increased sun exposure/Vitamin D levels [26–30]. It is likely that there are also unidentified intrinsic and environmental factors that affect AMH levels. These unknown factors deserve further investigation as they could be determinants for the wide ranges of AMH in women of similar age and may contribute to unexpectedly high AMH variability across the 6-month period for some subjects.

Despite the small sample size and information of yet unknown factors that affect AMH levels, this study has several strengths, namely, the careful screening process designed to exclude women with confounding factors including history of infertility, vaginal infections (other than HPV), and irregular menstrual cycles, which could bias case and control populations.

## 4. Conclusions

In summary, this study suggests that the recently reported increased time to pregnancy among women who have had cervical surgery is not the result of a LEEP-induced decrease in AMH, though the reason why there is delayed fertility in women treated with LEEP remains unknown [5]. Direct damage to the ovaries and ovarian reserve resulting from LEEP is doubtful. LEEP-induced endometriosis may inhibit joining of sperm and egg as would the destruction of mucus-producing cervical glands, which aid in sperm capitulation and subsequent fertilization [16, 31]. Additionally, direct or indirect physical damage to cervical tissue during LEEP could cause cervical stenosis, impair embryonic implantation, or result in an unfavorable microenvironment for pregnancy [15, 16, 32, 33]. Because half a million women of reproductive age are treated with LEEP each year in the United States alone, future research is needed to investigate the underlying causes for delayed time to pregnancy with LEEP [5].

## Supplementary Material

Supplemental Figure 1. Flow chart of the LEEP study subject recruitment process. A total of 112 participants completed the study. There were 63 qualifying subjects who required a LEEP procedure (cases: CIN2/3) and 49 subjects who did not require cervical surgery (controls: CIN2). A subset of the enrolled women (19 LEEP and 28 No LEEP controls) also consented to provide blood samples at baseline and at follow-up visits for future analysis. For the current study we selected all 19 available cases and 19 age-matched controls. One case-control pair was eliminated because of an approximately 300% increase in AMH levels from baseline to follow-up measurements.

## Figures and Tables

**Table 1:** AMH descriptive statistics.

	Baseline					Follow-up					*P* ^a^	% mean decrease (BL − FU)^c^
	*N*	Mean (SD)	Med.	Min.	Max.	*N*	Mean (SD)	Med.	Min.	Max.		
*AMH levels: all subjects *												
	36	3.56 (2.0)	3.19	0.73	7.73	36	3.07 (1.9)	2.66	0.66	8.82	0.009	13.76
*AMH levels by LEEP status *												
LEEP	18	3.02 (1.6)	2.81	1.05	6.55	18	2.66 (1.6)	2.19	0.82	5.76	0.07	11.92
No LEEP	18	4.09 (2.3)	3.32	0.73	7.73	18	3.48 (2.1)	3.22	0.66	8.82	0.06	14.91
*P* ^b^	0.11					0.2						

Abbreviations: AMH: anti-Müllerian hormone; LEEP: loop electrosurgical procedure.

^a^Paired *t*-test for baseline versus follow-up AMH levels (difference between the means).

^b^Analysis of variance to test for difference between mean at baseline or at follow-up for LEEP versus No LEEP.

^c^Baseline mean AMH value minus follow-up mean AMH value.

Med.: median; Min.: minimum; Max.: maximum.

**Table 2:** Percent change in AMH levels associated with crude and adjusted odds of LEEP, Iowa.

	LEEP *n* (%)	No LEEP *n* (%)
Continuous AMH	18 (100)	18 (100)
Percent change		
No change^a^	3 (16.7)	4 (22.2)
Increase	3 (16.7)	5 (27.8)
Decrease	12 (66.7)	9 (50.0)

Abbreviations: AMH: anti-Müllerian hormone; LEEP: loop electrosurgical excision procedure.

^a^No change is equivalent to an AMH change of ±9.9%.
