# Supplementary figures and images for: Does Loop Electrosurgical Excision Procedure of the Uterine Cervix Affect Anti-Müllerian Hormone Levels?
Source: Biomed Res Int. 2014 Feb 23;2014:875438. doi: 10.1155/2014/875438 (PMC3953513; doi:10.1155/2014/875438)

Supplemental Figure 1. Flow chart of the LEEP study subject recruitment process

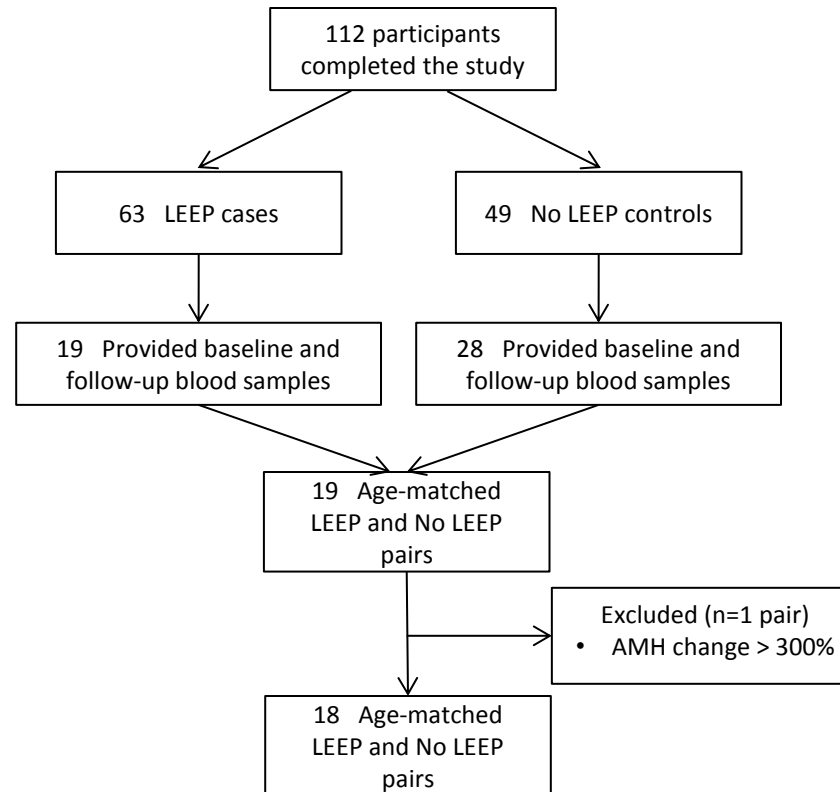

Supplement: Supplementary file 1 — Supplemental Figure 1. Flow chart of the LEEP study subject recruitment process. A total of 112 participants completed the study. There were 63 qualifying subjects who required a LEEP procedure (cases: CIN2/3) and 49 subjects who did not require cervical surgery (controls: CIN2). A subset of the enrolled women (19 LEEP and 28 No LEEP controls) also consented to provide blood samples at baseline and at follow-up visits for future analysis. For the current study we selected all 19 available cases and 19 age-matched controls. One case-control pair was eliminated because of an approximately 300% increase in AMH levels from baseline to follow-up measurements. [file 875438.f1.pdf]
